# Integration of Inertial Sensors in a Lower Limb Robotic Exoskeleton

**DOI:** 10.3390/s22124559

**Published:** 2022-06-16

**Authors:** John Calle-Siguencia, Mauro Callejas-Cuervo, Sebastián García-Reino

**Affiliations:** 1 GIIB Research Department, Universidad Politécnica Salesiana, Cuenca 010102, Ecuador; jcalle@ups.edu.ec (J.C.-S.); sebastian.garciar@ucuenca.edu.ec (S.G.-R.); 2 Software Research Group, Engineering Department, Universidad Pedagógica y Tecnológica de Colombia, Tunja 150003, Colombia

**Keywords:** actuators, exoskeleton, inertial sensors, Imocap-GIS, motion cycle, UDP protocol, lower limb

## Abstract

Motion assistance exoskeletons are designed to support the joint movement of people who perform repetitive tasks that cause damage to their health. To guarantee motion accompaniment, the integration between sensors and actuators should ensure a near-zero delay between the signal acquisition and the actuator response. This study presents the integration of a platform based on Imocap-GIS inertial sensors, with a motion assistance exoskeleton that generates joint movement by means of Maxon motors and Harmonic drive reducers, where a near zero-lag is required for the gait accompaniment to be correct. The Imocap-GIS sensors acquire positional data from the user’s lower limbs and send the information through the UDP protocol to the CompactRio system, which constitutes a high-performance controller. These data are processed by the card and subsequently a control signal is sent to the motors that move the exoskeleton joints. Simulations of the proposed controller performance were conducted. The experimental results show that the motion accompaniment exhibits a delay of between 20 and 30 ms, and consequently, it may be stated that the integration between the exoskeleton and the sensors achieves a high efficiency. In this work, the integration between inertial sensors and an exoskeleton prototype has been proposed, where it is evident that the integration met the initial objective. In addition, the integration between the exoskeleton and IMOCAP is among the highest efficiency ranges of similar systems that are currently being developed, and the response lag that was obtained could be improved by means of the incorporation of complementary systems.

## 1. Introduction

The development of exoskeletons has experienced widespread acceptance due to the diversity of tasks that these systems may perform, such as motion assistance, strain reduction, and continuous rehabilitation, among others [1]. Projects for exoskeleton development start with an analysis of the difficulties people may have, such as those who work in a fixed position or perform repetitive motions, to establish the maximum period during which these people may remain in this position without experiencing health problems. Another topic of study is that of exoskeletons that improve the user’s physical capabilities and that address situations that require force or speed that is significantly greater than those a human being can generate. Medical exoskeletons are developed for the rehabilitation of patients who have lost their mobility or whose mobility is reduced due to physical damage they have suffered, and these exoskeletons seek to improve such conditions [2,3].

There are several exoskeletons that fulfil the function of rehabilitation or walking support. Those with the greatest technological impact are listed below. Indego is an exoskeleton that provides locomotive assistance to people with paraplegia due to a lesion on the spinal cord. It has two brushless DC motors, a control system that calculates the user’s centre of pressure and carries out the movement, and it has six degrees of freedom [4]. Arke is an exoskeleton that assists people with paraplegia. It has three degrees of freedom, one in the knee and two in the ankle. It can be controlled using a tablet. This exoskeleton is for rehabilitation purposes [5]. Atalante is a completely autonomous exoskeleton that simulates natural movement by offering 12 degrees of freedom, with motors in the hip, knee, and ankle, without requiring additional support products. It features intuitive non-joystick controls, using a sensor vest, and it is programmed to navigate through obstacles. It is one of the first dynamic walking robots to be marketed in series, exclusively in the field of rehabilitation, and it is proposed as the initial option for walking, providing adaptive or partial assistance, as it is a lighter exoskeleton [6]. HAL-3 (hybrid auxiliary limb) is a voluntary driven exoskeleton that can improve gait functions in case of spinal cord injury and stroke. This study aimed to assess the safety and effects of HAL-supported treadmill therapy on the walking function of patients with limb-girdle muscular dystrophy (LGMD) [7]. RewalkTM has the peculiarity of allowing the user to ascend and descend stairs, in addition to the functions of getting up, sitting down, standing up, turning, and walking. Its walking speed of 2.6 km/h is also notable. The device consists of a series of sensors that initiate a forward step if a forward tilt of the upper body is detected by the system. The movement starts from the bilateral motors arranged on the hip and knee. The battery is integrated into the pelvic belt so that an external backpack is not required for its portability, and so this exoskeleton can be used to assist in walking [8].

The Biomedical Engineering Research Group (GIIB) of the *Universidad Politécnica Salesiana* in Ecuador has been working on a lower limb exoskeleton, which provides assistance during the motion cycle, starting from a kinematic analysis of this cycle to validate the generated trajectory [9]. Sensors with different data acquisition techniques for lower limb exoskeletons are also studied to identify the exoskeletons with the greatest impact [10].

To generate the mobility of exoskeletons, several types of signal acquisition can be applied, each one focused on specific areas and movements of the human body. Among the most important and up-to-date signal acquisition technologies are the following: computer vision, electroencephalography (EEG), electromyography (EMG), and inertial sensors, among others. These methods have been developed in recent years and have had a significant impact on the science of biomedicine [3].

Computer vision has applications in all areas of engineering, including the field of exoskeletons. The exoskeleton obtains information about its environment through an RGB-D camera and extracts the characteristics of the floor surface that could affect its gait. Then, it makes decisions according to the characteristics of the environment, the state of the surroundings, any security restrictions, and finally, it provides the adequate length and height of the step to the parameterized gait pattern planning model to assist the user with walking [10].

With the acquisition of EEG signals, the principal difficulty of this approach is how to effectively interpret the movement of the subject and provide this information to the exoskeleton to achieve motion [11].

The following studies are related to EEG cueing. The study ‘Prediction of gait intention from pre-movement EEG signals: a feasibility study’ seeks to carry out a predictive methodology to detect the intention to start and stop a gait cycle by using EEG signals obtained before the event occurs, resulting in the possibility of predicting the intention of human movements exclusively from the EEG signal prior to the movement that will be applied in prosthetic systems and in real-world neurorehabilitation [12]. In addition, in the study ‘Gait compensatory mechanisms in unilateral transfemoral amputees’, the objective was to determine if the initiation of the gait in non-amputees can be predicted using data that would be available in prosthesis users on the prosthetic side, showing that the detection of gait initiation intention allows 130–260 ms for the control of a prosthesis [13].

EMG sensors are non-invasive. In the study by [14], an adaptive estimator based on EMG was proposed to obtain and update the model without calibrations and recalibrations. Both the simulation and the experiments indicate that the proposed estimator can adaptively predict the active torque of the subject’s joint and guarantee the exoskeleton’s precise movement control. In the study by [15], a gait was developed based on speed estimator neural networks and slope using electromyography signals (EMG) and mechanical signal sensors. The results of four healthy and two elderly subjects demonstrate that the EMG approach can reduce the error rate by 14.8% compared with models that only use mechanical sensors.

Another type of sensor that is incorporated into this category are joint position sensors, which can be goniometers, accelerometers, gyroscopes, magnetometers, and inertial measurement units (IMU), which usually provide important information, and above all, are portable devices that facilitate the use of the orthotic device.

The importance of the use of IMU sensors can be noted in [16] which uses these sensors to establish the gait cycle. In this way, the kinematic model is analyzed, and the proposed model allows for calculating the position of the human leg and actuator’s characteristic points. In addition, the research in [17] details a calibration method to place and align inertial sensors with segments of the human body, with the aim of measuring joint angles. The advantages of the proposed method, compared to other methods, include the quick and easy placement of the sensors [18]. The authors of [19] detail information about the plantar sensors, which can be used to implement strategies to recognize human movement and detect gait subphases, resulting in better control of the user’s gait.

All of these sensors offer the capacity for combination with artificial intelligence (AI) and to be able to predict the movements that the exoskeleton wearer will perform, thus training the controller [20].

The Software Research Group (GIS) of the *Universidad Pedagógica y Tecnológica de Colombia* has developed the Imocap-GIS system, which consists of a System-on-Chip (SoC) microprocessor that orchestrates the operation, information processing, and coordination of the tasks under execution; a connection interface for peripherals that enables the reconfiguration of the device for multiple applications; a mass storage unit, which provides local support for data collection; wireless communication with different operation modes; and an electric management system that provides versatility and greater autonomy [21].

The objective of this study was to integrate a platform based on Imocap-GIS inertial sensors with an exoskeleton for gait accompaniment that generates the movement of the joints with Maxon motors and harmonic drive reducers. Imocap-GIS sensors take the positional data of the user’s lower extremities and send the information through the UDP protocol to the COMPAQ RIO system, which is a high-performance controller. These data are processed inside the card and subsequently the control signal is emitted to the motors so that they move the joints of the exoskeleton. The response time of the actuators in the exoskeleton is measured and the delay in the data taken by the sensor is determined by comparing them with the response times of the actuator through a correlation of the curves obtained, using the Pearson and Lin coefficients.

## 2. Materials and Methods

### 2.1. Exoskeleton Analysis

The exoskeleton built by the GIIB research group was used in this project. The design began with an analysis of the motion curves at different speeds, which were obtained from the following works: “A public dataset of running biomechanics and the effects of running speed on lower extremity kinematics and kinetics” [22], and “Biomechanics and motor control of human movement” [23]. The experimental values were obtained by the group GIIB, whose facility was where this project was carried out. A comparative analysis was carried out using the data collected to obtain the similarity of the motion to the experimental data, and this was compared to the works that were analyzed. Once the data and the motion characteristic curves were obtained, the exoskeleton was designed. To guarantee functionality, aesthetics, and ergonomics, a virtual design was initially carried out using the Autodesk Inventor 2020 software to generate the corresponding drawings, and the Adams View software was used to validate the functionality [9].

**Figure 1:**
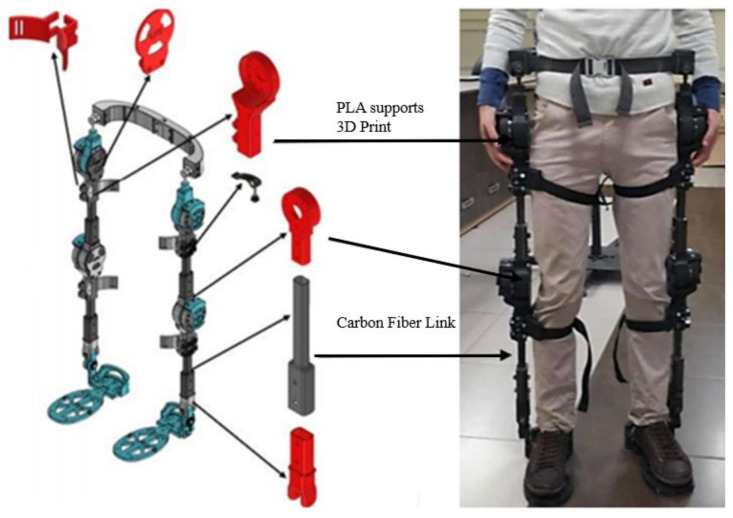
Exoskeleton developed by the GIIB.

The exoskeleton was designed based on the needs of people that execute repetitive activities or that need help with motion assistance. A biomechatronic design with twelve degrees of freedom (DOF) was carried out, which had six degrees of freedom in each leg, two at the hip, two at the knee, and two at the ankle, with the main objective being to ensure that the motion of the user is not affected by the mechanism and that the strain caused by the motion is reduced [9]. Important features, such as lightness, impact resistance, and fast couplings were also incorporated into the design. To obtain the aforementioned characteristics during manufacturing, carbon fibre was used for the coupling links between the foot, knee, and hip; in addition, some 3D printing was employed in some exoskeleton parts, such as the hip, motor mount, potentiometer mount, sole of the foot, etc. The exoskeleton geometry is tubular and has a weight of 18 pounds, and the fast coupling facilitates its adaptation to any user, as shown in Figure 1.

The security strategy was implemented in three ways. First, there was the programming phase. This generates a lock from the CompactRIO system when the sensor values emit an angle that is not established in the gait cycle, and thus the system does not perform said movement. As a second strategy, there is an emergency stop by means of a button, and currently, we are working on a mechanical system that does not allow the established gait angles to be exceeded.

### 2.2. Imocap-GIS System

The Imocap-GIS system developed by the GIS research group is used for signal acquisition. It has the advantage over other systems of having a compact and embedded design that enables the integration of all functional parts for collecting, processing, storing, and transmitting the bio-parameters without requiring a large size. In addition, Imocap-GIS supports up to 4 simultaneous Motion Processing Units (MPUs) based on inertial-magnetic technology [24].

**Figure 2:**
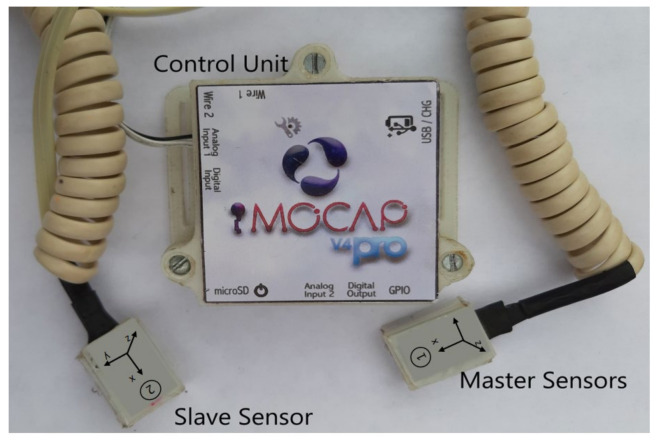
Imocap-GIS system.

The central processing system is based on the conceptualization of the system. The sensors that capture motion are placed on the thigh and knee segments, including the electrodes for capturing the muscle electromyographic activity, and are wirelessly connected to a central processing unit called Imocap-GIS; this system is visualized in Figure 2. In addition to providing the position of the joints accurately and real-time data acquired at a speed of 90 samples per second (samples/s) [25], the Imocap-GIS system also enables the operator to calculate the linear acceleration and angular speed of the movements in three pre-established axes (x, y, and z) in a precise and reliable way [26]. It is, therefore, a satisfactory tool to be integrated into the exoskeleton.

**Figure 3:**
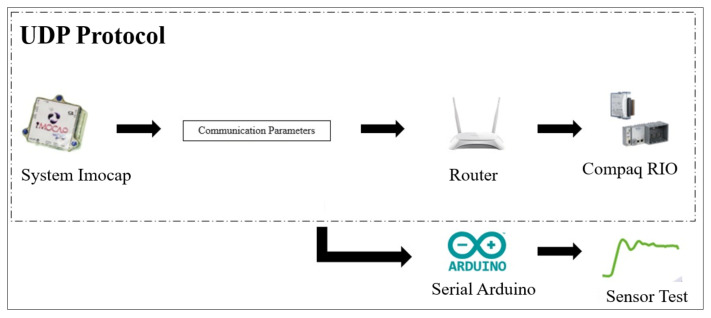
Connection diagram of the Imocap-GIS system.

The Imocap-GIS system connects with a CompactRio processing unit, which is a high-performance embedded controller, through a network to which both devices are connected, and data are sent by means of the UDP protocol. After setting up the necessary parameters, an Arduino serial monitor is used to verify via a USB port that all inertial sensors (IMUs) are sending data. Once the connection with the sensors has been established, they are placed at specific points on the lower limbs to detect user movements. This is crucial for the operation of the exoskeleton because there are points on the lower limbs at which data collection is not sufficiently stable or at which the information collected is of little relevance for the study; consequently, based on experiments, they are placed at points that provide more information about joint movements. Sensors are connected to the Arduino serial monitor through the USB port to be able to identify which sensors are active and send the correct data by means of the communication protocol implemented in the Imocap-GIS system, as shown in Figure 3.

**Figure 4:**
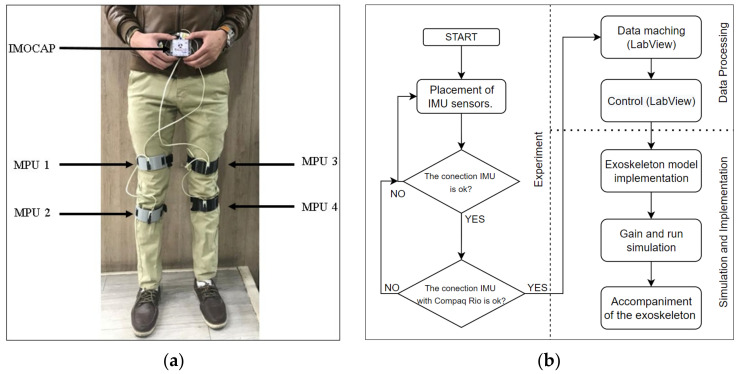
(**a**) Placement of the Imocap-GIS system for collecting information, (**b**) Flowchart of the experimental procedure.

Inertial sensors were placed as recommended in [10], which states that the IMUs should be aligned with the body segments of interest, evident in Figure 4a.

### 2.3. Integration of the Imocap-GIS System with the Exoskeleton

To integrate the exoskeleton with the Imocap-GIS, was necessary to realize a control system to enable interaction between system output, i.e., the motors of the exoskeleton, and the input, i.e., the signal from the inertial sensors of the Imocap-GIS system. A closed-loop control system was used to carry out this integration.

#### 2.3.1. Control System

**Figure 5:**
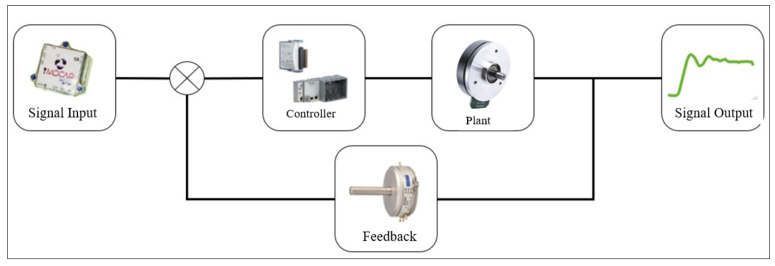
Control diagram of the Exoskeleton.

To build an appropriate controller it is necessary to establish the level with which you want to interact with the exoskeleton. For this, the more complex the interaction the more data inputs are required and therefore a greater data processing capacity. In the case of this study, a closed-loop system was chosen, which had a PD control (Figure 5) and as the main input there was an inertial-type signal and a control card with fast inputs and outputs that allowed for the real-time accompaniment of the walk.

**Figure 6:**
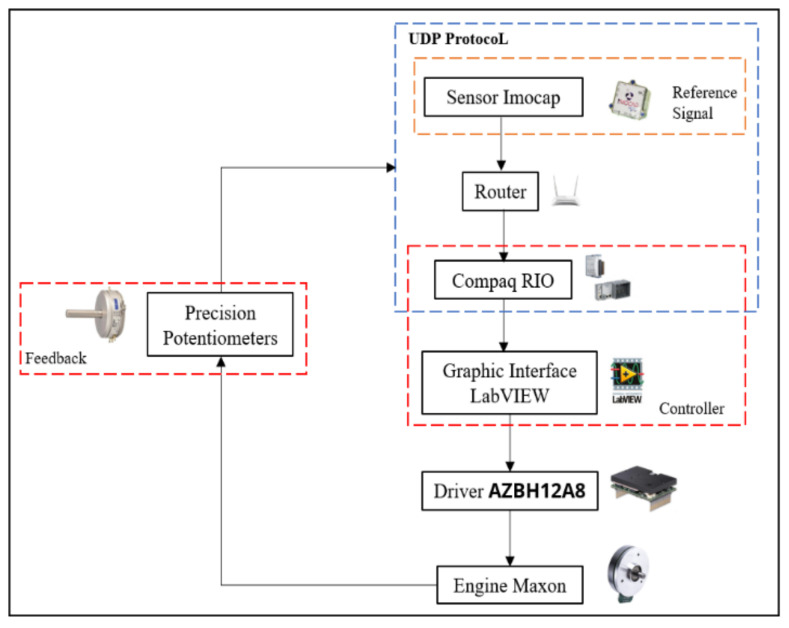
Block diagram of the integration.

To send data between the IMU sensors and the computer, the UDP communication protocol was used, where it was necessary to create an internal network that allows for communication between the Imocap Sensors and the Compaq RIO, in order to be able to obtain the reading of the sensors in LabVIEW, in addition to requiring digital input and output modules such as the NI 9201 that is connected to the precision potentiometers and NI 9263 for communication with the AZBH12A8 drivers that serve to control the Engine Maxon Series 2002939 motors, which move the exoskeleton joints. Thus, the total integration of the exoskeleton is achieved, as can be seen in Figure 6.

A user interface or front panel was built into LabVIEW, along with controls, indicators, and parameters. In this case, the controls are buttons for the user to interact with, such as start and stop, PID, and data acquisition, which are used to control the exoskeleton actuators. Graphic indicators were used to observe the motion changes produced by the sensors. The system has buttons for turning off the exoskeleton in the case of a failure, and as a safety feature, the exoskeleton returns to a resting position to prevent damage to the user; therefore, when the motors are completely turned off, they return to the zero or initial position, so that the user will return to the resting position.

##### Obtaining Exoskeleton Equations

**Figure 7:**
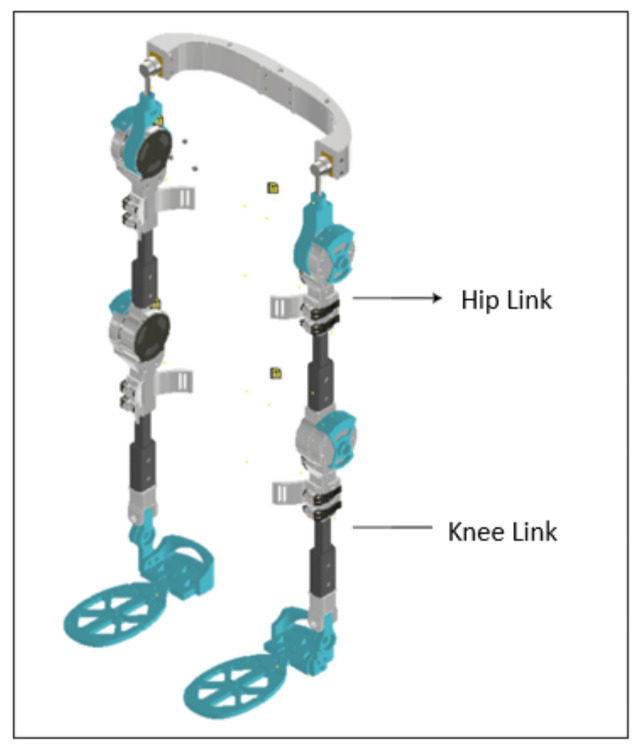
Exoskeleton Links.

The control of the exoskeleton is provided by the joints and links of the hip and knee. In these joints are the Maxon EC motors, so the first thing we define are the equations of the knee and hip based on the physical study of the conditions of the links presented in Figure 7.

Hip Link Equation [27]

(1) $$\frac{d}{dt}\left(\frac{\partial \;L}{\partial \dot{q_{i}}}\right)-\left(\frac{\partial \;L}{\partial q_{i}}\right)=T_{i}$$

where:

$q_{i}$ represents the generalized variables of the system;

$T_{i}$ represents the external inputs of the system;

L is the Lagrangian, which is calculated as follows,

(2) L=T−V 

where:

T represents the kinetic energy of the system;

V represents the potential energy of the system.

**Figure 8:**
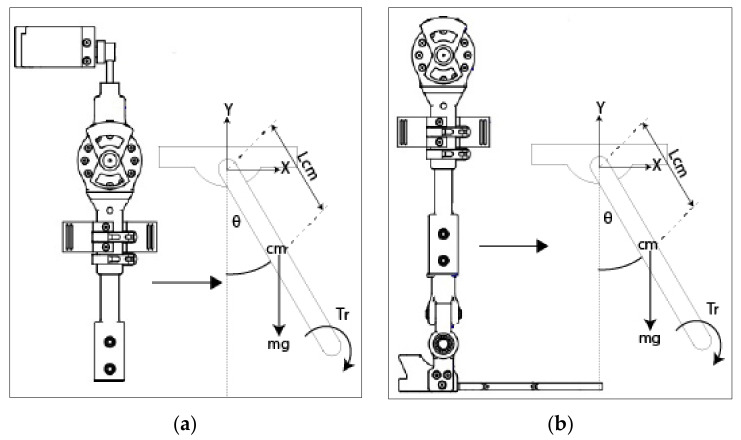
Diagram. (**a**) Hip Link Diagram (**b**) Knee Link Diagram.

The free-body diagram of the hip link is shown in Figure 8a.

(3) $$T=\frac{m_{c}*\;v_{c}{}^{2}}{2}+\frac{L_{cm}*\;{\dot{\theta }}_{c}{}^{2}}{2}$$

where:

$L_{cm}$ is the inertia of the link with respect to the centroid;

$m_{c}$ is the mass of the link;

$v_{c}$ is the velocity;

${\dot{\theta_{c}}}^{2}$ is the angular velocity.

(4) $$v_{c}=\left|\dot{r}\right|\;$$

In addition,

(5) $$r=L_{cm}*\sin\left(\theta_{c}\right)\;\hat{ı}-L_{cm}*\cos\left(\theta_{c}\right)\hat{ȷ}$$

(6) $$\left|\dot{r}\right|=L_{cm}*{\dot{\theta }}_{c}$$

so that

(7) $$T=\frac{m_{c}*\;{\left(L_{cm}*{\dot{\theta }}_{c}\right)}^{2}}{2}+\frac{L_{cm}*{{\dot{\theta }}_{c}}^{2}}{2}$$

(8) $$T=\frac{{{\dot{\theta }}_{c}}^{2}*\left(m_{c}*L_{cm}{}^{2}+L_{cm}\right)}{2}$$

(9) $$V=m_{c}*g*L_{cm}*\cos\left(\theta_{c}\right)$$

Thus, by replacing T and V in Equation (2) we have

(10) $$L=\frac{{{\dot{\theta }}_{c}}^{2}*\left(m_{c}*L_{cm}{}^{2}+L_{cm}\right)}{2}-m_{c}*g*L_{cm}*\cos\left(\theta_{c}\right)$$

Therefore

(11) $$\frac{d}{dt}\left(\frac{\partial \;L}{\partial \dot{qi}}\right)={\ddot{\theta }}_{c}*\left(m_{c}*L_{cm}{}^{2}+L_{cm}\right)$$

(12) $$\frac{\partial \;L}{\partial qi}=m_{c}*g\;*\;L_{cm}*\sin\left(\theta_{c}\right)\;$$

and

(13) $$T_{i}=T_{C}-T_{CR}-F_{a}$$

where:

$T_{C}$ is the input torque from the Maxon motor for the hip;

$T_{CR}$ is the knee load torque;

$F_{a}$ is the damping force.

Therefore

(14) $${\ddot{\theta }}_{c}*\left(m_{c}*L_{cm}{}^{2}+L_{cm}\right)-m_{c}*g\;*\;L_{cm}*\sin\left(\theta_{c}\right)=T_{C}-T_{CR}-F_{a}$$

Starting from the fact that the exoskeleton is a damped harmonic oscillator system, and this has a non-conservative force that can be assumed as a function proportional to the speed and is considered a generalized force for the exoskeleton equation, it is established that **F***_a_* is:

(15) $$F_{a}=\frac{1}{2}b{{\dot{\theta }}_{c}}^{2}$$

Knee Link Equation

The free-body diagram of the knee link is shown in Figure 8b.

Based on Equations (1)–(3), v can be obtained, which is the translational speed of the link and is given by:

(16) $$v_{R}=\left|\dot{r}\right|\;$$

where:

v is the speed of translation of the link given by

(17) $$r=-L_{cm}*\sin\left(\theta_{R}\right)\;\hat{ı}-L_{cm}*\cos\left(\theta_{R}\right)\hat{ȷ}$$

(18) $$\left|\dot{r}\right|=L_{cm}*{\dot{\theta }}_{R}$$

So that

(19) $$T=\frac{m_{R}*\;{\left(L_{cm}*{\dot{\theta }}_{R}\right)}^{2}}{2}+\frac{L_{cm}*{{\dot{\theta }}_{R}}^{2}}{2}$$

(20) $$T=\frac{{{\dot{\theta }}_{R}}^{2}*\left(m_{R}*L_{cm}{}^{2}+L_{cm}\right)}{2}$$

(21) $$V=-m_{R}*g*L_{cm}*\cos\left(\theta_{R}\right)$$

Thus, by replacing T and V in Equation (2) we have

(22) $$L=\frac{{{\dot{\theta }}_{R}}^{2}*\left(m_{R}*L_{cm}{}^{2}+L_{cm}\right)}{2}+m_{R}*g*L_{cm}*\cos\left(\theta_{R}\right)$$

So that

(23) $$\frac{d}{\mathrm{dt}}\left(\frac{\partial \;L}{\partial \dot{q_{i}}}\right)={\ddot{\theta }}_{R}*\left(m_{R}*L_{cm}{}^{2}+L_{cm}\right)$$

(24) $$\frac{\partial \;L}{\partial q_{i}}=-m_{R}*g\;*\;L_{cm}*\sin\left(\theta_{R}\right)$$

and

(25) $$T_{i}=T_{r}-F_{a}$$

where:

$T_{r}$ is the input torque from the Maxon motor;

$F_{a}$ is the damping force.

So that

(26) $${\ddot{\theta }}_{R}*\left(m_{R}*L_{cm}{}^{2}+L_{cm}\right)+m_{R}*g\;*\;L_{cm}*\sin\left(\theta_{R}\right)=T_{r}-F_{a}$$

and

(27) $$F_{a}=\frac{1}{2}b{{\dot{\theta }}_{R}}^{2}$$

Within Equations (16) and (27) the damping force is observed, which according to its formula is linearly proportional to the angular velocity. This consideration can cause drawbacks when modelling the controller of a real system so this makes it necessary to resort to system identification.

##### System Identification

**Figure 9:**
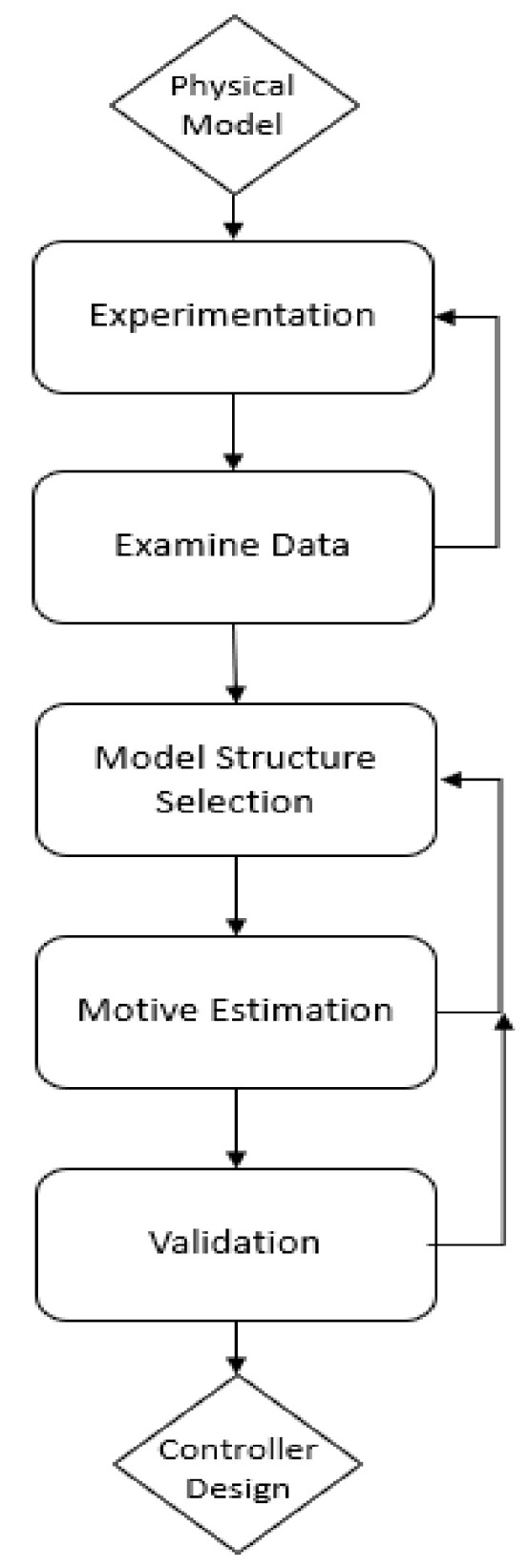
System Identification.

For the identification of a system, some steps must be followed to achieve the desired results, as shown in Figure 9.

System identification is the step prior to the design of the controller. These systems can be continuous or discrete either through classic techniques or state variables. A controller for the system was obtained using the system identification process.

Preliminary experiments provide the information necessary for the identification of the system. This experimentation is carried out based on an input and an output that are obtained from the programming developed in the LabView software. Later, they are processed within MATLAB, where the signal sampling time must be entered, and the structure of the model chosen must be entered to better fit the experimental conditions obtained.

The identified dynamic model can be used for simulation or error detection to later carry out the design of the controller.

##### Obtaining the Controller

When applying the systems’ identification algorithm described in the previous section, one of the most important points to arrive at the transfer function is the experimentation that must be carried out until a series of data are obtained that can be analyzed, and from these, an equation can be abstracted using MATLAB.

To validate that the data are correct, there is a verification system. This system measures the angular displacement of the joints by means of two methods of sensing the signal. The first method is by precision potentiometers placed in the joints that are read through the acquisition diagram presented above, and to verify that they are correct, the Imocap inertial sensors are used. The coincidence of these two signals under the Pearson coefficient is 0.9673, which makes the signal acceptable for abstracting the equation of the control from this data. This coefficient is obtained with the function corr2 within the MATLAB Software. Under these conditions, the MATLAB data are entered under the iddata command.

The next step is to enter all the identification conditions into MATLAB’s PID Tuner; the conditions under which the tests were performed. In this case, a square input signal of 1.5 V is entered with a period of 1 s and a duty cycle of 50%, and in this way, the outputs are obtained.

For the transfer function obtained in MATLAB to be as close as possible to reality, it is necessary to select the structure of the model that allows for the coincidence between the two signals to be high, and so an initial instance uses two complex poles, resulting in an equation that adapts 63.65% to the curve of experimental data. Thus, it was decided to change the model.

The next model that was chosen was a controller with two complex poles and a real one, and a zero is added to it, thus obtaining similarity percentages of 88.54% and 87.21% for the control of the hip and knee, respectively, under the Levenberg–Marquardt iteration method.

**Figure 10:**
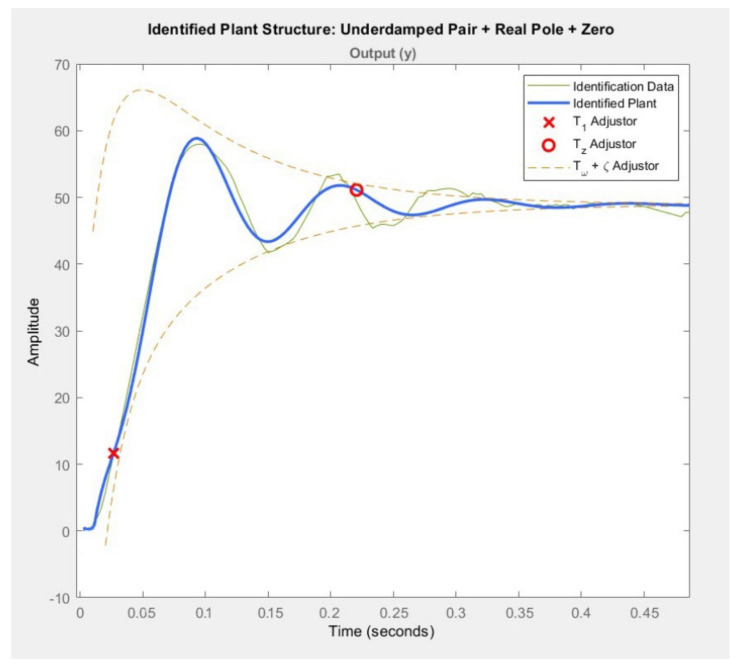
Controller of the Hip and Knee.

With these results, the equations of the hip and knee were obtained, which are shown in Figure 10, and whose structure behaves according to Equation (28).

(28) $$\frac{\theta \left(s\right)}{V_{in}\left(s\right)}=k\;*\left(\frac{\mathrm{Tz}*s+1}{\left(T1*s+1\right)\left({T\omega }^{2}{\;*\;s}^{2}+2*\zeta *T\omega *s+1\right)}\right)$$

where:

θ is the exit angle at which the joint will arrive;

$V_{in}$ is the input voltage to the motor that moves the joint.

The equations obtained through the data provided by PID Tuner and based on the model in Equation (28), for the hip and knee, respectively, are:

(29) $$\frac{\theta \left(s\right)}{V_{in}\left(s\right)}=\left(\frac{-301214.64571755*\left(s-18.536024764129\right)}{\left(s+55.4415922\right)*\left(s^{2}+22.235810*s+3090.1955046\right)}\right)$$

(30) $$\frac{\theta \left(s\right)}{V_{in}\left(s\right)}=\left(\frac{-84098.00926*\left(s-17.841531517065\right)}{\left(s+28.98718\right)*\left(s^{2}+20.3702822*s+2265.200\right)}\right)$$

**Figure 11:**
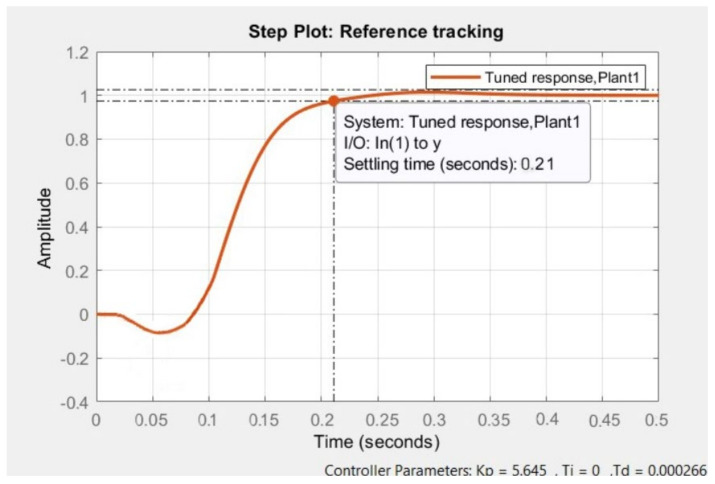
Control Conditions Obtained.

Then, these equations were interpreted in the PID Tuner PID adjustment to obtain a stable output without overshoots that stabilized in a time of around 0.2 s, so using the sliders, it was possible to adjust the step of the hip and knee to a time of 0.21 s and 0.23 s, respectively, as shown in Figure 11.

Once the adjustment process was carried out, the values for the PID control were obtained.

## 3. Results

After obtaining the data of the position of the exoskeleton links for slow, normal, and fast speeds, in consideration of study [20], this study was conducted with healthy patients and 20 gait cycles were obtained for each speed and were then analyzed using Pearson’s correlation coefficient and the Lin concordance coefficient.

Before entering the data in MATLAB, it was necessary to determine the delay that exists between the reference signal and the output signal from the actuators, and thus the overshoot (in degrees) of each sample was analyzed.

**Figure 12:**
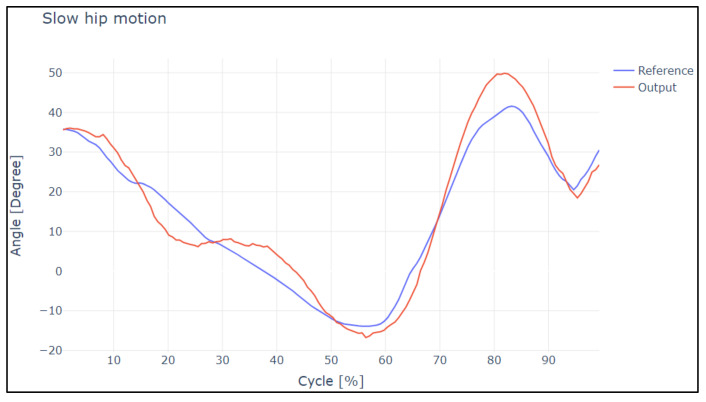
Reference curves vs. output during slow hip motion.

**Figure 13:**
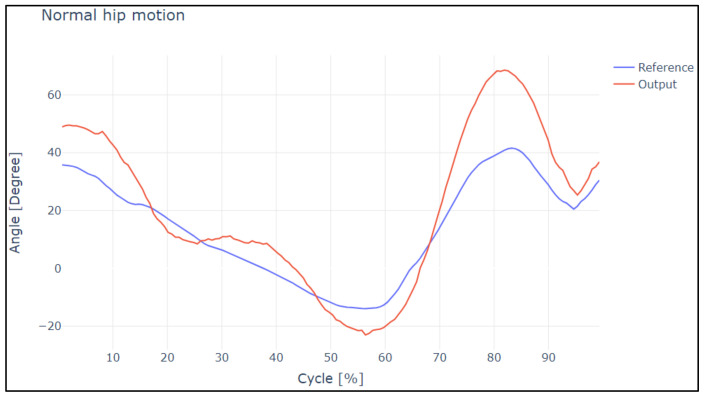
Reference curves vs. output during normal hip motion.

Figure 12 and Figure 13 show the overshoot of the slow and normal gait of the hip. Analyzing this image, it is evident that in a slow gait, the output reaches the reference satisfactorily; it has a 15.98% rate of change between both curves. These points were analyzed under a correlational study.

**Figure 14:**
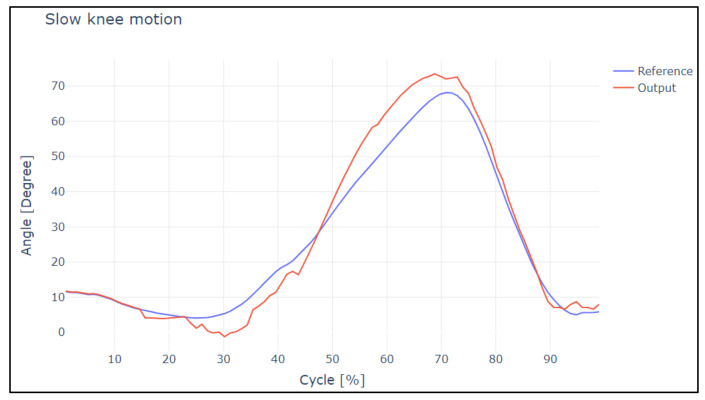
Reference curves vs. output during slow knee motion.

**Figure 15:**
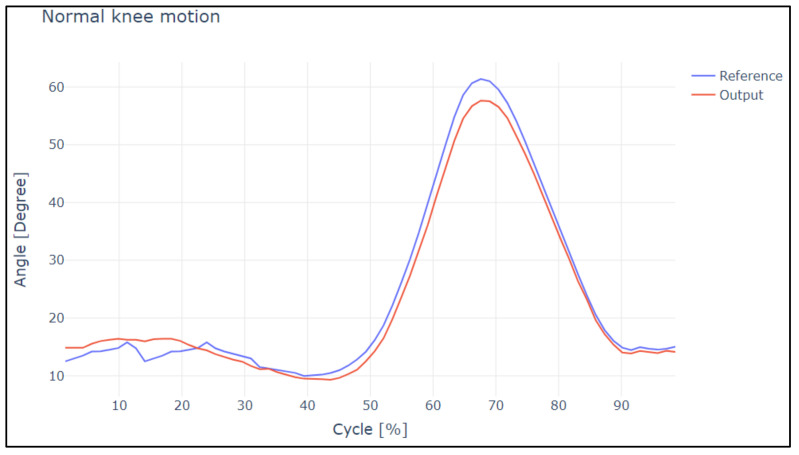
Reference curves vs. output during normal knee motion.

In Figure 14 and Figure 15, the overshoot of the slow and normal gaits of the knee is displayed. Analyzing this image, it is evident that the in slow gait, the output reaches the reference satisfactorily; it has a rate of change between both curves of 10.98%. This means that for the difference between both curves, and in the case of normal walking, there are small overshoots, and it has a rate of change between both curves of 16.98%. These points were analyzed under a correlational study.

In the case of slow and normal gaits in the hip and knee, it is evident that the gaits have a delay value of 20 and 30 ms, respectively, in addition to the values obtained by Pearson’s coefficient and Lin’s concordance coefficient; thus, we can say that they are satisfactory.

For fast motion, the change in the reference position is significantly abrupt, and thus the position of the links does not reach the reference value.

To study the correlation and similarity of the curves, the data are entered considering an average time lag of 20–30 ms; the obtained results are presented in Table 1.

Table 2 presents the correlation coefficients obtained in the similarity study of the curves for slow and normal motion of the knee joint.

## 4. Discussion

The integration of the exoskeleton with a portable alternative data acquisition system known as Imocap-GIS was evaluated by analyzing its motion. In this study, the integration of the IMU sensors was performed to guarantee the accompaniment of the exoskeleton with the movements of the individual, as opposed to study [16] where it was used to acquire the curves of the gait cycle and thus to be able to obtain a kinematic analysis for the characterization of the actuators.

The correlation results between the curves of the sensors and the exoskeleton output, using Pearson’s coefficient, reached an average of 0.93, i.e., a very high positive correlation, thus guaranteeing that the device will not limit the movements of the joints. In addition, analyzing it along with Lin’s concordance coefficient, which showed results greater than 0.90, we can say that it has a moderate degree of concordance. In this way, we guarantee that the device will not limit the movements of the joints. It is evident that the integration between the exoskeleton and the Imocap-GIS system is satisfactory.

The values obtained with slow knee joint motion are the following: at a speed from 0.25 to 0.5 m/s, a similarity of 0.9532 and a concordance coefficient of 0.9620; in the hip joint at the same speed, a similarity value of 0.944 and a concordance coefficient of 0.9881.

The values obtained with normal knee joint motion are the following: at a speed from 1 to 1.25 m/s, a similarity of 0.8721 and a concordance coefficient of 0.9220; in the hip joint at the same speed, a similarity value of 0.87.65 and a concordance coefficient of 0.9125.

The values obtained during fast motion with a speed from 2 to 2.25 m/s in the different joints did not follow user motion.

In [28], 10 gait cycles were carried out, wherein gait speeds ranging between 0.8 and 1.5 m/s and values of 0.9445 PCC and 0.8722 CCC were obtained. In [2], a similarity value of 10.57% between both curves was obtained; this study was carried out in real time and obtained approximately 6000 samples. In [29], the tests were carried out with ten healthy subjects to evaluate the precision of the proposed algorithm, and the results showed that the error of similarity in the hip was 7.7%; in the knee, it was 13.1%; and in the ankle, it was 6.8%. In [30], the test was carried out with five healthy subjects, obtaining an average latency of 638.97 ms and an average precision of 92.8% with IMU sensors and force sensors. In [31], 10 gait cycles were performed and the delay time was found to be 31.4 ms. Considering the cited studies, we can compare and analyze our values; we performed 20 gait cycles, obtaining the following values: an average Pearson coefficient of 0.9116 and an average Lin concordance coefficient of 0.9461. When compared with the values attained by Liu, we can say that our study had a better approximation between the curves. In addition, our similarity value between both curves was between 10% and 16%. When compared to the study by Chen and Yi, we can say that we are within the same range. If we compare our latency time of 20 to 30 ms, with respect to the study by Patzer, who obtained an average latency of 638.97 ms, we can say that our value is lower, but with respect to Kim, we obtained a similar value (See Table 3).

## 5. Conclusions

The efficient integration of sensors to guarantee the accompaniment of the exoskeleton with the movements of the individual is a task that requires high precision. If the movements of the parts of the exoskeleton generate significant delays, the person will quickly experience fatigue, and the exoskeleton will not fulfil its objective.

The exoskeleton developed by the Biomedical Engineering Research Group of the *Universidad Politécnica Salesiana in Ecuador* satisfactorily meets the DOF and ergonomics required for motion accompaniment; in addition, it is lightweight, weighing 18 lbs, and it is built with materials that guarantee its functionality and resistance. The Imocap-GIS system developed by the Software Research Group of the *Universidad Pedagógica y Tecnológica de Colombia*, with its compact and embedded design, enables the integration of all the functional parts to collect, process, store, and transmit the bio-parameters. The integration obtained between the exoskeleton and the Imocap is among the highest efficiency ranges of similar systems currently under development, and the obtained response delay of between 20 ms and 30 ms could be improved by incorporating supplementary systems.

## Figures and Tables

**Table 1:** Analysis of slow and normal hip motion.

Analysis	Pearson Coefficient	Concordance Coefficient
Slow Hip Motion		
Reference values—Output values	0.9447	0.9881
Normal Hip Motion		
Reference values—Output values	0.8765	0.9220

**Table 2:** Analysis of slow and normal knee motion.

Analysis	Pearson Coefficient	Concordance Coefficient
Slow Knee Motion		
Reference values—Output values	0.9532	0.9620
Normal Knee Motion		
Reference values—Output values	0.8721	0.9125

**Table 3:** Analysis of the results.

Article	Error between Curves	Response Delay	Velocity	Samples
This Document	15.98%	30 ms	0.25 to 0.5 m/s	20
	10.56%	30 ms	1 to 1.25 m/s	20
Chen	10.57%	145.12 ms	0.75 to 1.5 m/s	800
Yi	7.7%	22.5 ms	1 to 1.5 m/s	1000
	13.1%			
	6.8%			
Patzer	7.2%	638.97 ms	No Information	500
Kim	No Information	31.4 ms	0.8 to 1.5 m/s	20

## Data Availability

Not applicable.

## Abbreviations

EEG	(Electroencephalography)
EMG	(Electromyography)
UDP	(User datagram protocol)
SoC	(System-on-Chip)
GIIB	(Grupo de Investigación de Ingeniería Biomédica)
GIS	(Software Research Group)
IMU	(Inertial measurement units)
DOF	(Degree of freedom)
